# Quantity Over Quality: How the Rise in Quality Measures is Not Producing Quality Results

**DOI:** 10.1007/s11606-015-3278-6

**Published:** 2015-03-24

**Authors:** Michele L. Esposito, Harry P. Selker, Deeb N. Salem

**Affiliations:** Department of Medicine, Tufts Medical Center, Boston, MA USA

**Keywords:** quality improvement, performance measurement, medical errors, patient safety, Medicaid

## Abstract

Over the past decade, quality measures (QMs) have been implemented nationally in order to establish standards aimed at improving the quality of care. With the expansion of their role in the Affordable Care Act and pay-for-performance, QMs have had an increasingly significant impact on clinical practice. However, adverse patient outcomes have resulted from adherence to some previously promulgated performance measures. Several of these QMs with unintended consequences, including the initiation of perioperative beta-blockers in noncardiac surgery and intensive insulin therapy for critically ill patients, were instituted as QMs years before large randomized trials ultimately refuted their use. The future of quality care should emphasize the importance of evidence-based, peer-reviewed measures.

## INTRODUCTION

Over the past decade, quality measures (QMs) have assumed a crucial role in the healthcare landscape. With the advent of pay-for-performance and public reporting of hospital and physician adherence to quality guidelines, the development of safe, practical QMs is more important than ever. The Affordable Care Act (ACA) now mandates that the Centers for Medicare and Medicaid Services (CMS) increase the scope of pay-for-performance nationally. As part of this new legislation, value-based purchasing expands the role of pay-for-performance in quality improvement. Hospitals will now be offered incentive payments derived from a list of 25 different QMs.^1^ Yet despite advances in healthcare quality improvement practices, not all QMs have proven to be of value. This is particularly the case when new data emerge that countervail the evidence on which the QM was originally based. There are multiple QMs that have fallen short of their intended objectives or that have even led to patient harm (see Table 1). Given the potential for adverse consequences, the conversion of guidelines into performance measures should not occur without adequate high-quality evidence. In this discussion, we examine two QMs that have had widespread effects on patient care, and that may have led to increased mortality: perioperative beta-blockers and glucose control in critically ill patients. We also explore the use of patient safety indicators and reduced 30-day readmission rates, which have an increasing role in the assessment of hospital quality and compensation.

**Table 1:** Complications Reported from Studies Evaluating Quality Measures in Clinical Practice

Quality measure/indicator examined	Reported complications
Perioperative beta-blockers^2^	Increased mortality, risk of stroke and hypotension; decreased risk of nonfatal myocardial infarction
Intensive insulin therapy in critically ill patients^3^^,^ 4	Increased hypoglycemic episodes, increased mortality
Preemptive antibiotics for suspected community-acquired pneumonia^5^	No association between early antibiotics and outcomes
Blood pressure control in chronic kidney disease^6^	Increased mortality rates with lower diastolic pressures
Patient satisfaction in surgical care^7^^–^ 9	No association with hospital compliance with surgical quality measures
Prophylactic antibiotics for major surgical procedures^10^	No correlation with surgical site infection rates
Heart failure performance measures^11^	Not correlated with rehospitalization or 60–90-day post-discharge mortality
Length of hospital stay^12^	Not positively correlated with quality of care
30-day readmissions^13^^,^ 14	Multiple factors that lead to readmission, <20 % deemed preventable
Venous thromboembolism^15^	Limited utility from surveillance bias
Hospital-acquired pressure ulcers^16^	Difference in administrative vs surveillance incidence
Patient safety indicators^17^^–^ 21	Unable to assess preventable events, low positive predictive value

## PERIOPERATIVE BETA-BLOCKERS

Worldwide, 100 million patients undergo noncardiac surgery every year. It is estimated that 10-40 % of these procedures are complicated by a major adverse cardiac event.^22^ First introduced into the literature in 1973, perioperative beta-blockers (PBB) were suggested as a preoperative maneuver to reduce the mortality of patients undergoing noncardiac surgery.^23^ However, it was not until publication of the Dutch Echocardiographic Cardiac Risk Evaluation Applying Stress Echo (DECREASE) trials that PBB became a commonly employed metric of quality.

The DECREASE I trial, published in 1999 by a team led by Dutch researcher Don Poldermans, was halted prior to completion due to a reported overwhelming survival benefit seen in the beta-blocker arm.^24^ After the results of DECREASE I were reported, the Agency for Healthcare Research and Quality (AHRQ) identified PBB as one of the “clear opportunities for safety improvement;” the National Quality Forum put PBB on its list of *30 Safe Practices for Better Healthcare*; and the Physician Consortium and the Surgical Care Improvement Project designated PBB as a QM.^25^ Poldermans continued to publish new data as part of the DECREASE trials, notably DECREASE IV, which demonstrated an additional survival benefit with the use of PBB in intermediate-risk patients.^26^

In 2008, the results of POISE, the largest randomized controlled trial (RCT) to have been reported at that time, with over 8,000 participants, found a significant increase in mortality among patients randomized to beta-blockers versus placebo.^2^ Despite these new data, both the European Society of Cardiology (ESC) and the American College of Cardiology Foundation/American Heart Association (ACCF/AHA) joint guidelines continued to recommend PBB, with the ESC citing it as a class I recommendation.^27^

In September 2012, the Netherlands-based Erasmus Medical Center released the final report of an investigation of suspected research misconduct in the DECREASE trials.^28^ After thorough examination, the committee concluded that there were multiple instances of scientific misconduct represented in the collection, analysis, and representation of source data.

Subsequent to this announcement, a meta-analysis published in 2013 with consolidated outcomes of over 10,000 randomized participants showed that initiation of PBB before surgery caused a significant increase in mortality, whereas the DECREASE data had shown a non-significant reduction in mortality.^29^ It was only after additional RCTs had countered the results of DECREASE that, in 2014, the ESC guidelines recommended against the initiation of PBB for patients undergoing low- to intermediate-risk noncardiac surgery.^30^

## GLUCOSE CONTROL IN CRITICALLY ILL PATIENTS

Stress-induced hyperglycemia is a known complication among critically ill patients. Multiple studies have shown hyperglycemia to be linked to increased mortality in both diabetic and non-diabetic cohorts. An initial trial in the surgical intensive care unit showed a mortality benefit with intensive insulin therapy (IIT).^31^ Based on this RCT in surgical patients, strict glucose control was recommended as a QM in the Surgical Care Improvement Program.^32^ Citing grade A evidence, the American Association of Clinical Endocrinologists and the American Diabetes Association issued recommendations in 2007 and 2008, respectively, advising tight glucose control in the critically ill population.^33^^,^ 34 Thus, although originally recommended for surgical patients in intensive care, the QM also began to be used among patients in non-surgical intensive care units. In 2009, the NICE-SUGAR trial, which enrolled both surgical and non-surgical patients, showed increased rather than decreased mortality rates and more frequent hypoglycemic episodes with intensive glucose control at 90 days.^3^ A meta-analysis published in 2011 showed no clear mortality benefit with IIT; once again, IIT was associated with increased risk of hypoglycemia.^4^ That same year, the American College of Physicians released guidelines stating that IIT should not be used in critically ill patients, either with or without diabetes.^35^

## PATIENT SAFETY INDICATORS

Patient safety indicators (PSIs) were released by AHRQ in March 2003 to identify post-surgical or post-procedural complications of inpatient care using billing information as a screening mechanism. PSIs are playing a greater role in QMs, as U.S. News recently announced that patient safety will be a more heavily weighted score in its Best Hospitals ranking system.^36^ Of the 17 total PSIs, eight comprise 10 % of the overall score in the new algorithm for this popular ranking system, double the 5 % contribution to the score the previous year.

The current list of PSIs is extensive, including complications such as iatrogenic pneumothorax, postoperative sepsis, and postoperative hemorrhage or hematoma. However, a 2008 study by Isaac and Jha found largely poor or inverse correlations between several PSIs and other hospital quality standards.^17^ These PSIs were poorly correlated with other process metrics and in-hospital mortality. Multiple other studies have investigated various PSIs. One looked at the validity of 12 different PSIs and found moderate positive predictive values for most of the quality indicators in detecting true safety events, concluding that these indicators required revision before their use as pay-for-performance measures.^18^ Similar conclusions were formed in several other studies validating PSIs for postoperative pulmonary embolus and VTE, iatrogenic pneumothorax, accidental puncture and laceration, central venous catheter-related bloodstream infections, and postoperative respiratory failure.^19^^–^ 21 Despite the lack of meaningful correlation between these PSIs and other process-of-care metrics, at this writing, they continue to have a role in hospital compensation and public reporting.

## THIRTY-DAY READMISSIONS

On October 1, 2012, CMS began penalizing hospitals for higher readmission rates for heart failure, acute myocardial infarction, and pneumonia as part of the Hospital Readmissions Reduction Program (HRRP). Of note, about 25 % of all patients discharged after admission for heart failure are readmitted within this 30-day bracket.^37^ However, it is unclear whether the factors that influence hospital readmission rates are inherently beyond their control. A study by Joynt and Jha found that many of these factors were related to characteristics of the patient population and their community resources, such as poverty, mental illness, social support, and good access to care,^38^ yet no specific targets for improvement were found that would be successful in preventing future readmissions. In an Ontario study, investigators found that less than one-fifth of readmissions within 6 months were actually preventable.^39^ Based on the pattern of reimbursement cuts, one study has shown that large teaching hospitals and safety-net hospitals are most likely to be penalized, suggesting that higher readmission rates might be due to lower socioeconomic status and greater case complexity.^38^

Currently the scope of HRRP is increasing. For the next fiscal year, CMS has expanded its list of diagnoses that will incur a readmission penalty to include acute myocardial infarction, heart failure, pneumonia, chronic obstructive pulmonary disease, and total hip or knee arthroplasty; in addition, the maximum Medicare reimbursement reduction will increase from 2 % to 3 %.^39^

## DISCUSSION

The translation of guidelines into performance measures requires a discriminating approach. Guidelines are a series of expert recommendations based on scientific evidence, and QMs are the next step in the evolutionary process of influencing care. Much more than a recommendation, a performance measure is a mandate that affects fiscal compensation and public reputational standing. QMs influence patient care, institutional compliance, and organizational financial well-being. In order to bolster the validity of performance measures and reinforce their impact, attention needs to be focused not only on developing guidelines, but also on the conversion of these guidelines to QMs. This evolution must be based on the best and most clinically relevant evidence available.

Clinical practice guidelines represent the consensus of experts, ideally based on evidence. However, the transformation of guidelines into QMs and pay-for-performance measures may antedate and even preempt the collection of high-quality evidence. In an analysis of class I ACC/AHA guideline recommendations over a period of several years, revisions in recommendations were found most commonly among those not verified by multiple RCTs.^40^ Clinicians may prematurely endorse and propagate new guidelines based on “consensus validity,” when the consensus process itself—sometimes at the expense of other, more relevant criteria—determines the content and/or adoption of new guidelines.^41^ Guidelines can also be influenced by panel members and their societies’ interests, and are not always just the result of evidence or good clinical judgment. The recent scandal involving a member of the National Quality Forum, who was allegedly paid over $11 million to endorse measures that would be financially favorable to his sponsor, serves to reinforce this serious concern.^42^

Future QMs need to be evaluated in a more rigorous and evidence-based manner. With the initiation of value-based purchasing as part of the new ACA standards, a greater number of QMs will now be proposed and marketed on a national platform and will require constant vigilant examination. To reduce the burden of unsafe practice, QMs must be subject to stringent scientific scrutiny, and hence must allow for peer-reviewed pay-for-performance (PR-P4P). A process such as PR-P4P is a directive with the goal of optimal fusion of economic incentives and patient-centered care. Although the past decade will undoubtedly be remembered for the gains made in patient safety and quality of care, continued improvement will require greater attention to the principles of scientific rigor in assessing measures of patient care quality and safety.
